# Co‐Anchoring of Engineered Immunogen and Immunostimulatory Cytokines to Alum Promotes Enhanced‐Humoral Immunity

**DOI:** 10.1002/adtp.202100235

**Published:** 2022-04-07

**Authors:** Jason Y. H. Chang, Yash Agarwal, Kristen A. Rodrigues, Noor Momin, Kaiyuan Ni, Benjamin J. Read, Tyson J. Moyer, Naveen K. Mehta, Murillo Silva, Heikyung Suh, Mariane B. Melo, K. Dane Wittrup, Darrell J. Irvine

**Affiliations:** ^1^ Koch Institute for Integrative Cancer Research Massachusetts Institute of Technology 500 Main Street Cambridge MA 02142 USA; ^2^ Ragon Institute of Massachusetts General Hospital Massachusetts Institute of Technology and Harvard University Cambridge MA 02139 USA; ^3^ Department of Biological Engineering Massachusetts Institute of Technology Cambridge MA 02139 USA; ^4^ Harvard‐MIT Health Sciences and Technology Program Institute for Medical Engineering and Science Massachusetts Institute of Technology Cambridge MA 02139 USA; ^5^ Consortium for HIV/AIDS Vaccine Development The Scripps Research Institute La Jolla CA 92037 USA; ^6^ Department of Chemical Engineering Massachusetts Institute of Technology Cambridge MA 02139 USA; ^7^ Department of Materials Science and Engineering Massachusetts Institute of Technology Cambridge MA 02139 USA; ^8^ Howard Hughes Medical Institute Chevy Chase MD 20815 USA

**Keywords:** aluminum hydroxide, germinal centers, humoral response, immunostimulatory cytokines

## Abstract

Protein antigens are often combined with aluminum hydroxide (alum), the most commonly used adjuvant in licensed vaccines; yet the immunogenicity of alum‐adjuvanted vaccines leaves much room for improvement. Here, the authors demonstrate a strategy for codelivering an immunostimulatory cytokine, the interleukin IL‐21, with an engineered outer domain (eOD) human immunodeficiency virus gp120 Env immunogen eOD, bound together to alum to bolster the humoral immune response. In this approach, the immunogen and cytokine are co‐anchored to alum particles via a short phosphoserine (pSer) peptide linker, promoting stable binding to alum and sustained bioavailability following injection. pSer‐modified eOD and IL‐21 promote enhanced lymphatic drainage and lead to accumulation of the vaccine in B cell follicles in the draining lymph nodes. This in turn promotes enhanced T follicular helper cell priming and robust germinal center responses as well as increased antigen‐specific serum IgG titers. This is a general strategy for codelivery of immunostimulatory cytokine with immunogens providing a facile approach to modulate T cell priming and GC reactions toward enhanced protective immunity using the most common clinical vaccine adjuvant.

## Graphical abstract

Alum is the most common clinical vaccine adjuvant; however, alum often elicits weak immune responses when combined with subunit protein antigens. Here, the authors develop a strategy to boost the immune response by co‐anchoring vaccine antigens and the cytokine interleukin‐21 (IL‐21) to alum particles; IL‐21 acts as a molecular adjuvant to enhance the antibody response against the antigen.

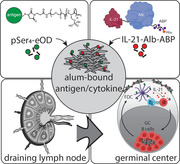

## 1 Introduction

The generation of neutralizing antibodies by vaccines occurs in germinal centers (GCs), specialized regions in the follicles of lymph nodes where B cells proliferate and mutate their antibody genes to evolve antibodies with high affinity to target antigens.^[^ ^1^, ^2^, ^3^, ^4^ ^]^ The GC reaction is regulated by cell‐cell interactions and a variety of secreted factors. Cytokines such as interleukin (IL)‐4 and IL‐21 play critical roles in directing B cells to enter GCs and promote their proliferation, survival, differentiation, and somatic hypermutation within GCs.^[^ ^5^, ^6^, ^7^ ^]^ These cytokines are secreted by T follicular helper (T_FH_) cells to provide crucial feedback signals during GC reaction kinetics via T cell‐dependent B cell activation. Interestingly, IL‐21 plays a pivotal role in GC formation in response to T cell‐dependent antigens, manifested as an autocrine feedback loop established by T_FH_ cells driving IL‐21 production and IL‐21R expression. T_FH_ cells depend on IL‐21 signaling for cell growth, survival, and differentiation, and their function directly impacts GC formation and IgG1 production.^[^ ^8^, ^9^, ^10^ ^]^ Furthermore, IL‐21 also plays a direct role in GC initiation, providing guiding signals for early B cell differentiation and migration of perifollicular pre‐GC B cells toward the intrafollicular regions of the GC.^[^ ^5^, ^11^ ^]^

The importance of these signaling molecules for directing the GC response suggest they might have utility as molecular adjuvants during immunizations, although it is important to consider the temporal relationship between antigen and cytokine delivery, as the relative timing of these cues may dramatically impact the humoral immune response. Prior studies of DNA vaccines have demonstrated that immunization with plasmid DNA encoding human immunodeficiency virus (HIV)‐1 Env gp120 antigen together with recombinant cytokines (including IL‐2, IL‐4, and IL‐21) was largely ineffective in altering the immune response due to rapid clearance of the cytokine into the bloodstream.^[^ ^12^, ^13^, ^14^, ^15^ ^]^ This is likely at least in part a consequence of the low molar mass of most cytokines, which due to their small size will primarily exit tissue by passing into the blood vasculature rather than trafficking into lymphatic vessels.^[^ ^16^ ^]^ Consistent with this idea, DNA vaccines encoding cytokines fused with an antibody Fc domain were much more effective.^[^ ^12^, ^13^, ^14^ ^]^ The duration of cytokine availability in draining lymph nodes (dLNs) is likely also important in eliciting beneficial effects on the ongoing immune response, and this may also be an important factor in the beneficial effects of cytokine delivery in these studies. However, it is unclear if sustained expression of a cytokine from plasmid DNA would be a safe approach in humans, especially when considering systemic exposure and off‐target effects.

We recently described an approach to enhance the efficacy of protein subunit vaccines by employing the most common clinical vaccine adjuvant, alum (aluminum hydroxide). Protein immunogens were site‐specifically modified with phosphoserine (pSer) peptide linkers, which undergo a ligand exchange reaction with the surface of alum particles to achieve stable binding tuned by the valency of the pSer peptide sequence.^[^ ^17^ ^]^ pSer‐mediated anchoring of immunogens to alum promoted prolonged antigen delivery to lymph nodes (LNs) via slow dispersal of alum particles from the immunization site, and enhanced B cell triggering at the single cell level as B cells encountered antigens multivalently arrayed on alum particles in the LN.

We hypothesized that phosphate‐mediated binding to alum could also facilitate the co‐anchoring of complementary molecular adjuvants, such as immunostimulatory cytokines, to further shape the ensuing humoral immune response. Here we explored the codelivery of pSer‐antigen with pSer‐tagged IL‐21, as a critical molecular cue to kick‐start the immune response and initiate the early stages of GC formation.^[^ ^6^, ^11^, ^18^ ^]^ Using the same design principles, we engineered an IL‐21 fusion protein containing a C‐terminal phosphorylated alum binding peptide (ABP) moiety linked to albumin (Alb) as a physical spacer (IL‐21‐Alb‐ABP hereafter), to enable alum binding and facilitate sustained antigen/cytokine codelivery to LNs to synergistically drive the humoral response.^[^ ^19^ ^]^ As a model immunogen, we employed an HIV envelope immunogen that recently entered clinical trials, eOD‐GT8 (engineered Outer Domain‐Germline Targeting 8, abbreviated as eOD hereafter). We characterized binding of IL‐21‐Alb‐ABP and pSer‐tagged eOD with alum particles in vitro, assessed the trafficking of this vaccine in vivo, and assessed impacts of alum‐anchored cytokine delivery on the GC reaction and serum antibody titers in mice.

In this study, we show that 1) pSer‐tagged eOD and IL‐21‐Alb‐ABP fusion proteins exhibit better retention on alum in vitro, resulting in slower in vivo clearance up to 1‐month post immunization; 2) the alum‐bound antigen and cytokine induced a strong GC response by targeting the follicles; and 3) the alum‐bound antigen and cytokine enhanced both the T_FH_ and GC response, promoting robust anti‐eOD serum IgG titers.

## 2 Results and Discussion

### 2.1 Phosphoserine‐Modification of Immunogens and Immunostimulatory Cytokines Promotes Enhanced Binding to Alum and Slower Clearance In Vivo

**Figure 1:**
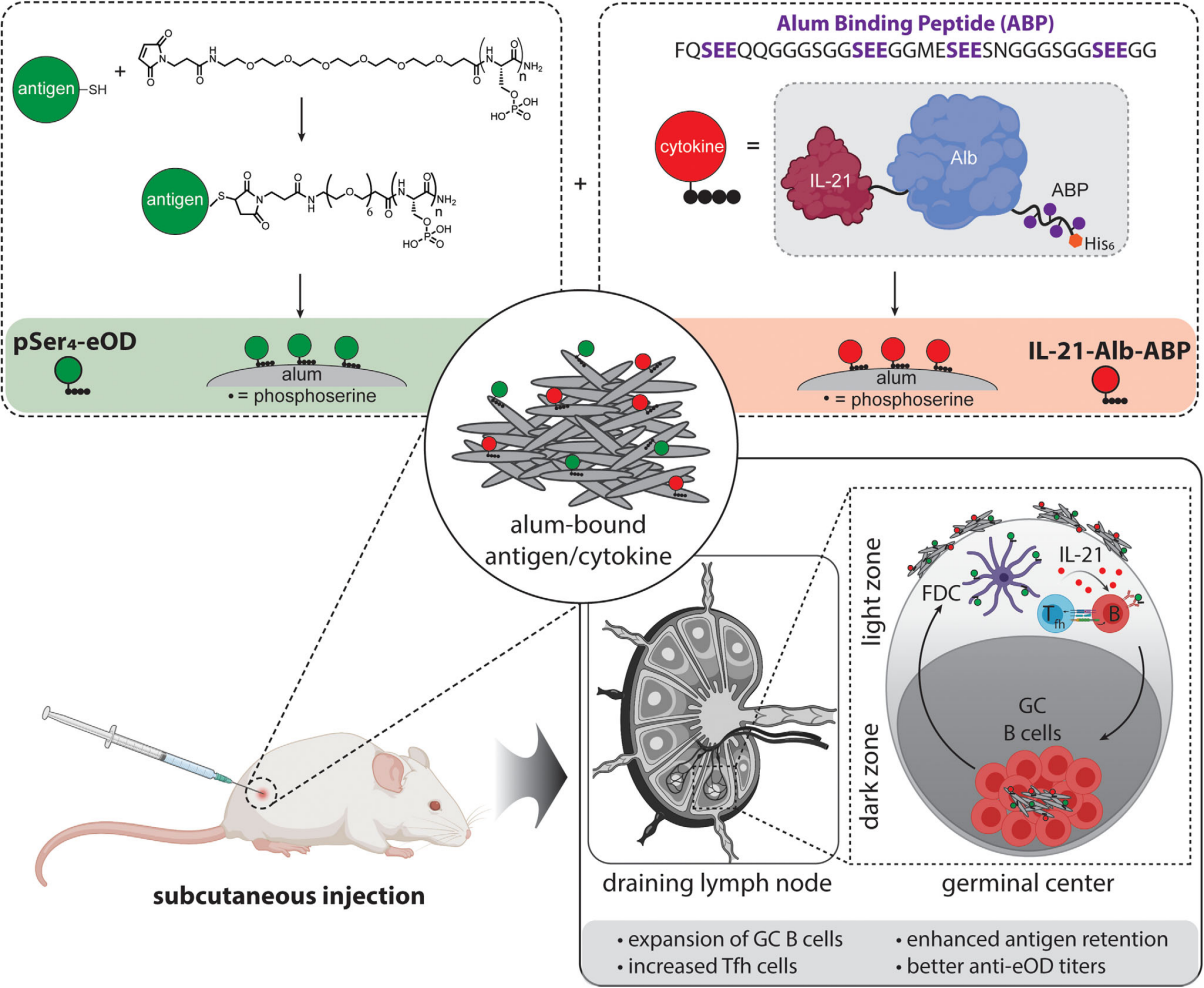
Schematic illustration of phosphoserine (pSer_4_) peptide‐modified eOD protein and IL‐21‐Alb‐ABP fusion protein bound to alum for codisplay of both the antigen and immunostimulatory cytokines to the draining lymph node, to enhance humoral responses in vivo.

**Figure 2:**
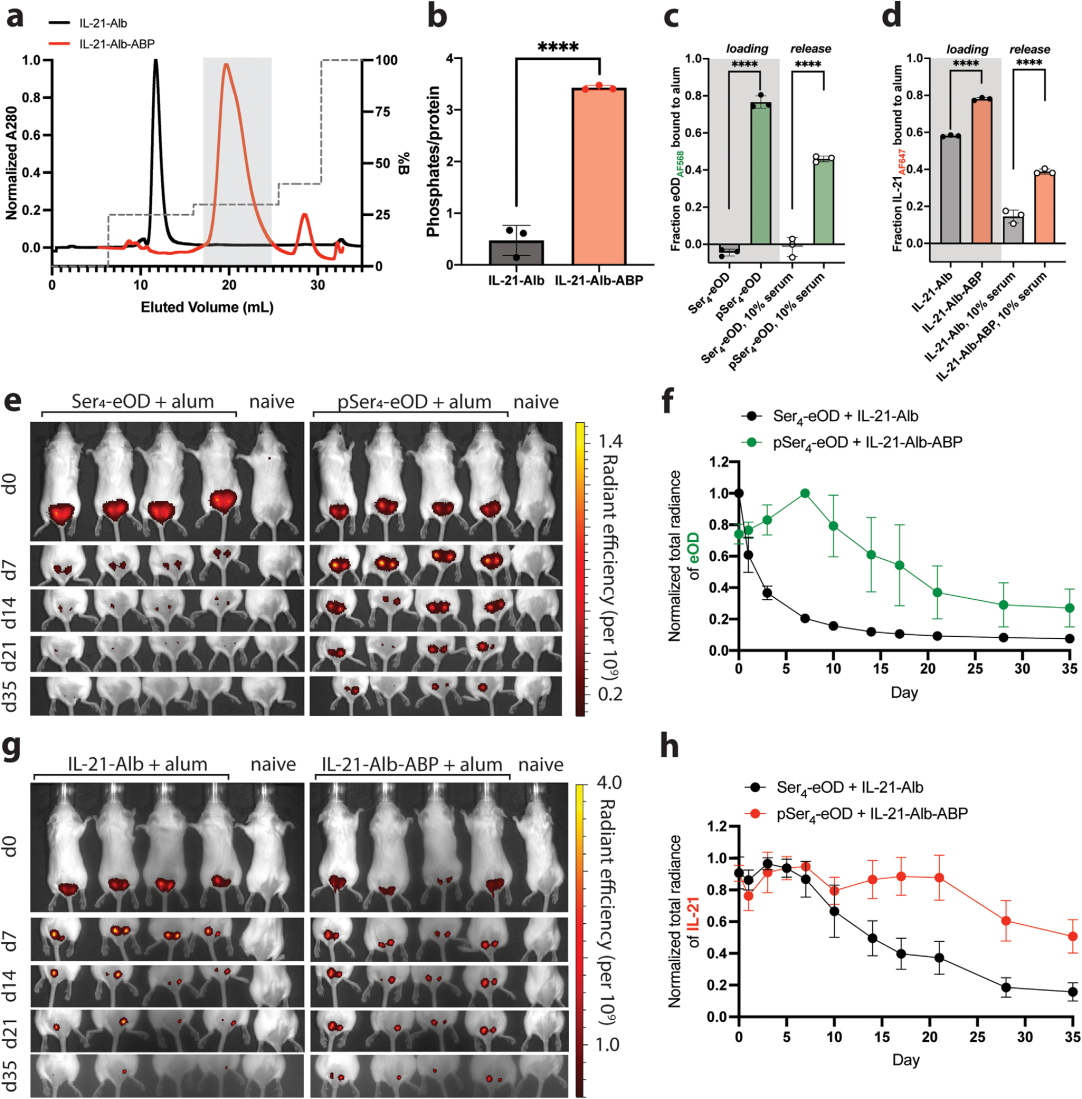
Codelivery of pSer‐conjugated eOD protein and IL‐21‐Alb‐ABP fusion protein via anchoring to aluminum hydroxide adjuvant. a) Representative trace of anion exchange chromatography of IL‐21‐Alb‐ABP (red trace) to separate phosphorylated cytokine compared to IL‐21‐Alb (non‐phosphorylated cytokine; black trace). Purified phosphorylated IL‐21‐Alb‐ABP peak indicated by the grey shaded box. b) Phosphorylation of IL‐21‐Alb‐ABP was determined with a malachite green assay to estimate the number of phosphoserine residues per protein. c,d) Fluorescently labeled eOD proteins with Alexa Fluor 568 (either Ser_4_‐ or pSer_4_‐conjugated; at 10 µg mL^−1^) and fluorescently labeled IL‐21 fusion proteins with Alexa Fluor 647 (either IL‐21‐Alb or IL‐21‐Alb‐ABP; at 10 µg mL^−1^) were mixed with Alhydrogel (100 µg mL^−1^) for 30 min, then incubated in PBS containing 10% mouse serum for 1 h, followed by fluorescence spectroscopy to measure protein remaining bound to alum. e–h) Fluorophore‐labeled Ser_4_‐eOD or pSer_4_‐eOD (5 µg of protein) and fluorophore‐labeled IL‐21‐Alb or IL‐21‐Alb‐ABP (30 µg of protein) were mixed with Alhydrogel (50 µg of alum), and injected s.c. in BALB/c mice (*n* = 4 animals per group) followed by longitudinal whole animal in vivo imaging system (IVIS) imaging of fluorescence at the injection sites. Representative whole animal images for eOD protein longitudinal monitoring are shown (e) and normalized total radiance from groups of animals over time (f). Representative whole animal images for IL‐21 fusion protein longitudinal monitoring are shown (g) and normalized total radiance from groups of animals over time (h). Statistical significance was determined by one‐way ANOVA followed by Tukey's post hoc test. ****p < 0.0001. Values plotted are means ± standard deviation.

The objective of this work was to test the concept that co‐tethering of a protein immunogen and recombinant cytokine to alum particles via pSer peptide anchors could be used to promote antibody responses by achieving sustained delivery of cytokine and antigen to draining LNs, promoting formation of an intranodal depot of the cytokine for sustained stimulation of B cells in developing GCs, and favoring colocalization of the cytokine payload with antigen for optimal stimulation of responding B cells (**Figure** **1**). The HIV Env antigen eOD is a ≈21.5 kDa gp120 engineered outer domain antigen designed to initiate priming of human B cells and promote CD4^+^‐binding site‐specific broadly neutralizing antibodies known as VRC01‐class antibodies.^[^ ^20^, ^21^, ^22^ ^]^ Following our recent report,^[^ ^17^ ^]^ we conjugated pSer_4_‐peptide linkers (containing four pSer residues, Figure 1 upper left) to an N‐terminal free cysteine on eOD via thiol‐maleimide coupling to aid the anchoring of the antigen to alum; serine (Ser_4_)‐peptide linkers were used as control linkers. Unlike eOD, IL‐21 contains multiple internal cysteines, and hence a similar approach based on site‐specific coupling of a solid phase‐synthesized pSer peptide using maleimide chemistry could not be used for the cytokine. To create phosphorylated IL‐21, we instead used a complementary strategy based on an in‐cell phosphorylation approach we recently developed:^[^ ^23^ ^]^ In preliminary studies, we found that the native mouse IL‐21 sequence expressed poorly in mammalian cells (similar issues are found with murine IL‐2). Thus to enhance expression yields, IL‐21 was fused at its C‐terminus with mouse serum Alb followed by a short ABP (Figure S1a,b, Supporting Information). The ABP contains several SXE motifs that are recognized by the kinase Fam20C and are phosphorylated during expression in cells that co‐express Fam20C (Figure 1 upper right and Figure S1b, Supporting Information).^[^ ^20^, ^21^, ^22^ ^]^ IL‐21‐Alb‐ABP was produced by co‐expressing secreted IL‐21‐Alb‐ABP with ER‐retained Fam20C in HEK293 cells, and purified from the culture supernatants using immobilized Ni‐NTA affinity chromatography, followed by anion exchange chromatography to isolate monomeric, phosphorylated protein (**Figure** **2**a and Figure S1c–e, Supporting Information). The purified protein was then run on a nonreducing SDS‐PAGE gel and a malachite green assay to confirm the molecular weight and number of phosphates per protein, respectively (Figure 2b). The potency of IL‐21‐Alb‐ABP in triggering proliferation of IL‐21‐responsive ANBL6 cells was similar to recombinant IL‐21 (Figure S1f, Supporting Information).

To evaluate binding of pSer_4_‐eOD and IL‐21‐Alb‐ABP to alum, both the antigen and cytokine were adsorbed onto alum separately for 30 min, then the fraction of protein that remained bound to alum was determined after a 30 min incubation in PBS, consistent with vaccine preparation. In buffer, ≈70–80% of pSer‐modified eOD was adsorbed to alum during incubation, whilst Ser‐modified eOD had negligible amounts of antigen bound to alum (Figure 2c “loading”). IL‐21‐Alb‐ABP also resulted in ≈70–80% of the protein adsorbed to alum (Figure 2d “loading”). TEM imaging of IL‐21‐Alb‐ABP‐loaded alum revealed no gross alterations in alum morphology following cytokine adsorption, and dynamic light scattering analysis of the alum particle size distribution showed only a slight upward shift in mean alum particle size following cytokine binding (Figure S2, Supporting Information). Note that this particle size represents the mean size of aggregates formed by individual nanoscale aluminum hydroxide crystals, which can disaggregate dynamically over time in vivo. The zeta potential of neat Alhydrogel as expected was positive (+22.8 ± 0.4 mV)), while loading of IL‐21‐Alb‐ABP led the zeta potential to become slightly negative (−5.99 ± 0.2 mV). Relatively high levels of IL‐21‐Alb lacking the ABP tag was also adsorbed to alum in the loading phase (≈50–60% protein bound to alum; Figure 2d). To test the stability of binding, the protein‐loaded alum samples were then incubated in buffer containing 10% mouse serum for 24 h at 37 °C on a tube rotator, and retention of bound protein was measured (Figure 2c‐d “release”). Approximately 50% of the adsorbed pSer_4_‐eOD and approximately 40% of IL‐21‐Alb‐ABP were still bound to alum following this serum exposure, whereas Ser_4_‐eOD and IL‐21‐Alb had much lower levels of retention after incubation (<1% and ≈15%, respectively). We next tested whether co‐adsorption with IL‐21‐Alb‐ABP affected binding of the eOD antigen to alum. As shown in Figure S3, Supporting Information, co‐adsorption of eOD on alum in the presence of IL‐21‐Alb or IL‐21‐Alb‐ABP did not meaningfully impact the initial loading in buffer or retention during serum incubation.

We next investigated the clearance and bioavailability of pSer‐modified cytokine and antigen compared to non‐alum‐binding control proteins when administered with alum in vivo (Figure 2e–h). To determine the clearance kinetics of the antigen and cytokine separately, the eOD antigens (either pSer_4_‐eOD or Ser_4_‐eOD) were labeled with Alexa Fluor‐750 dye, and the cytokines (either IL‐21‐Alb‐ABP or IL‐21‐Alb) were labeled with Alexa Fluor‐647 dye. The labeled vaccines were co‐adsorbed to alum and injected s.c. in BALB/c mice, and the fluorescence signal at the injection site was monitored over time by whole‐animal fluorescence imaging. Ser_4_‐eOD quickly cleared the injection site (≈40% of total signal remaining by day 3), whereas pSer_4_‐eOD signal persisted for >4 weeks (Figure 2e,f), similar to our previous findings with pSer_4_‐eOD adsorbed to alum.^[^ ^17^ ^]^ Consistent with the stronger alum adsorption of IL‐21‐Alb versus Ser_4_‐eOD observed in vitro, IL‐21‐Alb cleared slowly from the injection site over ≈4 weeks, while IL‐21‐Alb‐ABP cleared even more slowly than pSer_4_‐eOD, with ≈50–60% of total cytokine signal still remaining at the injection site at the terminal time point (day 35) (Figure 2g, h). Notably, the slow clearance kinetics observed for both IL‐21‐Alb and IL‐21‐Alb‐ABP reflected interactions with alum, as injection of the free proteins without alum led to rapid clearance from the injection site within ≈1 day (Figure S4, Supporting Information).

### 2.2 Alum‐Bound Immunogen and Cytokine Accumulates in Draining LNs and Localizes to B Cell Follicles

**Figure 3:**
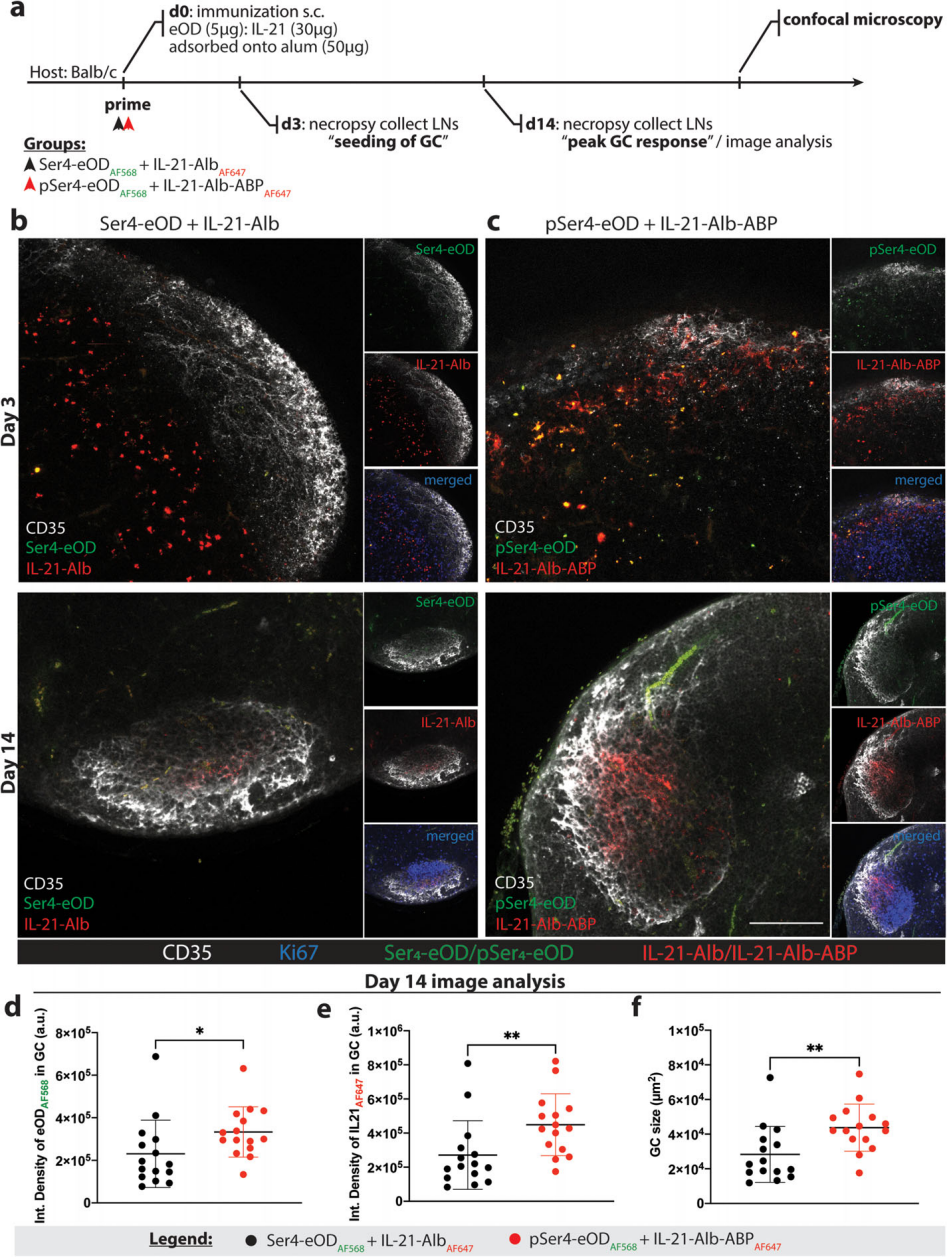
Codelivery of alum‐bound eOD and IL‐21 induces robust germinal center responses by targeting the FDC network. a) Schematic representation of the experimental design for antigen and cytokine trafficking to the draining lymph nodes. BALB/c mice (*n* = 3 mice per group) were immunized with fluorophore‐labeled Ser_4_‐eOD or pSer_4_‐eOD (Alexa Fluor‐568) and fluorophore‐labeled IL‐21‐Alb or IL‐21‐Alb‐ABP (Alexa Fluor‐647) and processed for immunohistochemistry and imaged via confocal microscopy for antigen and cytokine trafficking patterns and localization within B cell follicles. b,c) Representative confocal microscopy images of individual B cell follicles in draining lymph nodes illustrating antigen (green) and cytokine (red) trafficking to B cell follicles and display on the FDC network (day 3; top panels) and retention of antigen (Ser_4_‐eOD or pSer_4_‐eOD; green) and cytokine (IL‐21‐Alb or IL‐21‐Alb‐ABP; red) signal in the germinal center (day 14; bottom panels). FDCs and GCs were labeled with anti‐CD35 (white) and anti‐Ki67 (blue), respectively. d–f) The average size of GCs was measured and analyzed for colocalization of antigen and cytokines within the GCs on day 14. The total integrated fluorescence intensity for both the antigen and cytokine was quantified (*n* = 15 follicles per group sampled from six dLNs per group). Statistical significance was determined by two‐tailed Mann–Whitney *U*‐test. *p < 0.05; **p < 0.01. Values plotted are means ± standard deviation.

Next, we investigated whether the alum‐bound antigen and cytokine promoted improved LN trafficking and retention in B cell follicles. Here, we labeled the antigen with Alexa Fluor‐568 (either pSer_4_‐eOD or Ser_4_‐eOD) and the cytokine IL‐21 with Alexa Fluor‐647 (either IL‐21‐Alb‐ABP or IL‐21‐Alb), then immunized BALB/c mice subcutaneously. Draining inguinal LNs were harvested on days 3 or 14, times corresponding to the initial seeding of GCs and the peak GC response, respectively, and processed for confocal microscopy (**Figure** **3**a). Sections were stained with anti‐CD35 antibody to identify follicles and follicular dendritic cells (FDCs), while ongoing GCs were identified by staining with the proliferation marker Ki67. At day 3, the Ser_4_‐eOD:IL‐21‐Alb group had very little antigen signal (green) and IL‐21‐Alb (red) in the follicles as illustrated in Figure 3b (top panel). In contrast, the pSer_4_‐eOD:IL‐21‐Alb‐ABP group showed substantial colocalization of antigen and IL‐21‐Alb‐ABP in areas next to and overlapping the FDC network, and signs of the initial stages of GC formation based on Ki67 staining as illustrated in Figure 3c (top panel). By day 14, antigen was nearly undetectable in the Ser_4_‐eOD:IL‐21‐Alb group, and only low amounts of IL‐21‐Alb were observed, which was concentrated in Ki67+ GCs (Figure 3b, bottom panel). Strikingly, in the pSer_4_‐eOD:IL‐21‐Alb‐ABP group, substantial amounts of both antigen and cytokine signal were detected in the FDC network, though their colocalization was not as pronounced as at day 3 (Figure 3c; bottom panel). Interestingly, when we quantified the fluorescent intensity signals for both the antigen and cytokine localized to the FDC network, we found that the pSer‐modified proteins had greater overall signal retained in the follicles when compared to the control proteins (Figure 3d,e). Further, we measured the average size of GCs for the pSer_4_‐eOD:IL‐21‐Alb‐ABP group and found they were ≈1.5× larger than the GCs induced by Ser_4_‐eOD:IL‐21‐Alb immunization (Figure 3f). Together, these findings suggest that enhanced anchoring of antigen and IL‐21 together on alum promote larger GCs through enhanced follicular trafficking of the vaccine and sustained antigen and cytokine availability in the site of GC induction.

### 2.3 Co‐Anchoring of Both pSer‐Modified Antigen and Immunostimulatory Cytokine to Alum Amplifies Humoral Responses

**Figure 4:**
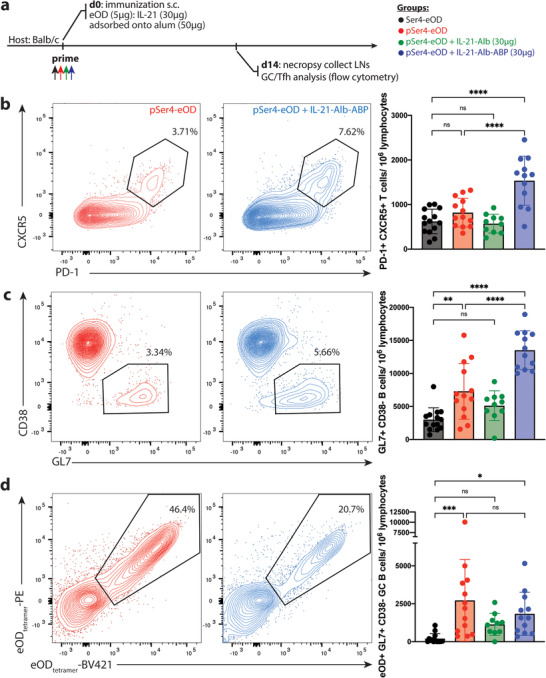
Alum‐bound pSer_4_‐conjugated eOD and IL21‐MSA‐ABP elicit potent germinal center responses and enhanced T_FH_ cells. a) BALB/c mice (*n* = 10–15 mice/group) were immunized s.c. with 5 µg of eOD (either Ser_4_‐ or pSer_4_‐modified affinity tags) and 50 µg of alum with or without 30µg of IL21 fusion protein (either IL‐21‐Alb or IL‐21‐Alb‐ABP). b–d) Germinal center (GC) and T follicular helper (T_FH_) responses were analyzed by flow cytometry on day 14. Shown are representative flow cytometry flow plots for T_FH_ cell count per 10^6^ lymphocytes (b), GC B cell count per 10^6^ lymphocytes (c), and eOD‐specific GC B cell count per 10^6^ lymphocytes (d). Data was pooled from three independent experiments. Statistical significance was determined by one‐way ANOVA followed by Tukey's post hoc test. ns, not significant; *p < 0.05; **p < 0.01; ***p < 0.001; ****p < 0.0001. Values plotted are means ± standard deviation.

We next evaluated the impact of alum‐bound IL‐21 delivery on the GC response by flow cytometry (Figure S5, Supporting Information). GC reactions are tightly governed by follicular helper T cells (T_FH_), and the magnitude of T_FH_ responses typically correlate with the number of B cells entering GCs.^[^ ^24^ ^]^ Thus, we first analyzed the number of T_FH_ cells generated following eOD/IL‐21 immunization in the presence or absence of alum binding moieties. pSer_4_‐eOD/IL‐21‐Alb‐ABP/alum immunization elicited an approximately twofold greater T_FH_ response over both the pSer_4_‐eOD and Ser_4_‐eOD groups (**Figure** **4**a,b). This enhanced T_FH_ expansion was not observed for immunization with IL‐21‐Alb lacking the ABP tag, suggesting that enhanced‐early colocalization of antigen and cytokine promoted by the ABP may be important for promoting the follicular helper T cell response.

Parallel to enhanced T_FH_ induction, phosphate‐anchoring of both antigen and cytokine anchoring to alum elicited the strongest total GC B cell response, with pSer_4_‐eOD:IL‐21‐Alb‐ABP immunization eliciting a nearly fivefold greater GC response than vaccination with non‐phosphorylated Ser_4_‐eOD/IL‐12‐Alb (Figure 4c). Interestingly, when comparing the effects of IL‐21‐Alb and IL‐21‐Alb‐ABP complexed with pSer_4_‐eOD onto alum, pSer_4_‐eOD:IL‐21‐Alb immunization (the IL‐21‐Alb fusion protein without pSer residues) did not elicit a statistically significant increase in GC responses. In fact, the pSer_4_‐eOD:IL‐21‐Alb group trended toward a slightly diminished GC B cell response when compared to pSer_4_‐eOD control group lacking any cytokine, although this did not reach statistical significance. These findings are reminiscent of prior studies of HIV gp120 DNA vaccine immunizations combined with IL2‐Ig fusion proteins, where a diminished immune response was elicited in the presence of the cytokine due to a misaligned temporal relationship between antigen and cytokine delivery.^[^ ^12^ ^]^ Here, only the alum‐anchored IL‐21‐Alb‐ABP fusion protein provided synergistic enhancement of the immune response during vaccinations.

Next, we examined whether this expansion of GC B cells translated to more antigen‐specific GC B cells. The frequency of eOD‐specific GC B cells was not statistically different for immunizations with pSer_4_‐eOD alone or combined with IL‐12‐Alb‐ABP, but each of these conditions induced much higher antigen‐specific GC responses than the control Ser_4_‐eOD vaccination (Figure 4d). We hypothesize that the IL‐12‐Alb‐ABP did not elicit a greater antigen‐specific GC B cell population due to sustained IL‐21 signaling promoting exit from the GC for responding B cells, as IL‐21 has been shown to promote GC B cells differentiation into long‐lived plasma cells and memory B cells.^[^ ^5^, ^25^ ^]^

**Figure 5:**
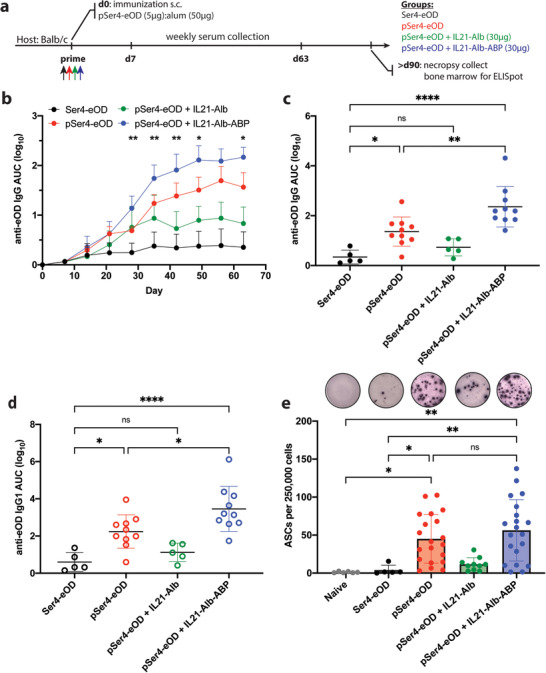
Codelivery of pSer_4_‐anchored immunogen and immunostimulatory cytokine on alum elicits enhanced humoral immune responses. a) BALB/c mice (*n* = 5–10 mice/group were pooled from two independent experiments) were immunized s.c. with 5 µg of eOD (either Ser_4_‐ or pSer_4_‐modified affinity tags) and 50 µg of alum with or without 30 µg of IL‐21 fusion protein (either IL‐21‐Alb or IL‐21‐Alb‐ABP). b–d) Serum IgG titers were analyzed weekly over time by ELISA. Serum IgG titers tracked longitudinally post immunization (b). Serum IgG titers (log AUC) for week 6 are shown (c). Serum IgG1 antibody isotype titers (log AUC) were also assessed for week 6 (d). eOD‐specific antibody secreting cells (ASCs) in the bone marrow were assayed by ELISPOT 3 months after immunizations (e). Data was pooled from three independent experiments. Statistical significance was determined by one‐way ANOVA followed by Tukey's post hoc test. ns, not significant; *p < 0.05; **p < 0.01; ****p < 0.0001. Values plotted are means ± standard deviation.

We next assessed how combined antigen/IL‐21 delivery impacted downstream serum antibody responses. We immunized BALB/c mice with pSer_4_‐eOD:IL‐21‐Alb‐ABP, pSer_4_‐eOD:IL‐21‐Alb, pSer_4_‐eOD, or Ser_4_‐eOD combined with alum and monitored serum IgG titers followed by a terminal bone marrow isolation for antigen‐specific ELISPOT (**Figure** **5**a). pSer_4_‐eOD:IL‐21‐Alb‐ABP with alum immunizations elicited robust IgG responses, with all animals seroconverting by 3‐weeks post‐prime and total IgG levels approximately twofold greater than pSer_4_‐eOD and approximately fourfold greater than Ser_4_‐eOD group, respectively, 6‐weeks post‐prime and these responses persisted to increase up to 9‐weeks post‐prime before declining (Figure 5b,c). Notably, immunization with pSer_4_‐eOD combined with IL‐21‐Alb elicited reduced titers compared to pSer_4_‐eOD alone. Serum titers were predominantly IgG1 isotype with minimal contributions from IgG2a and IgG2b, (Figure 5d and Figure S6, Supporting Information, respectively), which is consistent with the antibody profile expected from alum immunization in mice. ELISPOT analysis of bone marrow plasma cells 3 months after a single immunization showed a 14‐fold increase in antigen‐specific plasma cells elicited by immunizations with pSer_4_‐eOD compared to Ser_4_‐eOD immunization (Figure 5e).^[^ ^17^ ^]^ Interestingly, the number of antigen‐specific plasma cells were only slightly increased for the pSer_4_‐eOD:IL‐21‐Alb‐ABP group when compared to pSer_4_‐eOD group, and this did not reach statistical significance. Matching the observation in serum IgG levels, co‐immunization with IL‐21‐Alb actually blunted the plasma cell response. We hypothesize that while phosphorylated IL‐21 fusion showed an enhanced retention in GCs (Figure 3e), non‐phosphorylated IL‐21 may disperse more widely in the dLN and act on other cell types (e.g., T cells) in a manner that is counter productive to the humoral response—this will require future work to further elucidate. Overall, these results indicate that pSer_4_‐eOD:IL‐21‐Alb‐ABP anchored to alum promote enhanced‐humoral immune responses, through the expansion of GC B cells, T_FH_ cells, and the enhanced‐serum IgG responses.

## 3 Conclusion

In conclusion, we have demonstrated a strategy to enhance the immunogenicity of alum‐adjuvanted subunit vaccines by codelivering immunostimulatory cytokines, such as IL‐21, which plays a crucial role in the formation and regulation of GC reactions. The sustained delivery of both the HIV Env immunogen eOD and immunostimulatory cytokine IL‐21 helped synergistically promote a robust humoral immune response. Our findings show that alum‐bound proteins promote slow lymphatic drainage from the injection site, together with enhanced accumulation of both immunogen and cytokine in the FDC network over time, aiding the expansion of GC B cells and T_FH_ cells, and in turn better IgG responses. These data align with other recent studies suggesting that prolonging antigen exposure during vaccination over 2–4 weeks may be optimal for eliciting amplified antibody responses without immunosuppression.^[^ ^17^, ^18^, ^19^, ^20^, ^21^, ^22^, ^23^, ^24^, ^25^, ^26^, ^27^, ^28^, ^29^ ^]^ This strategy of codelivery of immunostimulatory cytokines with immunogens can pave the way toward better vaccine designs by tailoring cytokine kinetics to modulate GC reactions leading to enhanced humoral immunity.

## 4 Experimental Section

### Animals and Immunizations

Experiments and handling of mice were conducted under federal, state, and local guidelines under an Institutional Animal Care and Use Committee approved protocol (IACUC protocol number 0717‐076‐20). Female 6–8‐week‐old BALB/c mice were purchased from the Jackson Laboratory (stock no. 000651). Immunizations were prepared by mixing 5 µg of antigen, 30 µg of cytokine‐fusion protein, and 50 µg of aluminum hydroxide adjuvant (Alhydrogel or alum; InvivoGen) in 100 µL of sterile phosphate‐buffered saline per mouse. Antigen and cytokine were loaded onto alum sequentially for 30 min each at 25 °C on a tube rotator prior to immunization. Mice were immunized subcutaneously (s.c.) at the tail base with 50 µL on each side.

### Immunogen Synthesis and pSer Linker Conjugation

eOD‐GT8 gp120 antigens were synthesized as previously described.^[^ ^17^, ^20^ ^]^ Briefly, eOD with an N‐terminal cysteine was expressed in HEK cells and purified on a Nickel affinity column followed by size exclusion chromatography on a Superdex 75 10/300 column (GE Healthcare). Cys‐eOD was modified with pSer‐linkers using a thiol‐maleimide reaction. Both pSer and Ser peptide linkers were synthesized in‐house via solid phase peptide synthesis, as previously described.^[^ ^17^, ^30^ ^]^ In a solution of PBS, protein at 1 mg mL^−1^ was reduced for 15 min in ten equivalents of tris(2‐carboxyethyl)phosphine hydrochloride (TCEP, Thermo Fisher), and then TCEP was removed by centrifugal filtration using Amicon Ultra Centrifugal spin filters (10 kDa MWCO; Millipore Sigma). eOD at 1 mg mL^−1^ was reacted with 2 molar equivalents of maleimide‐pSer for 18 h at 4 °C in pH 7.4 PBS. Proteins were then separated from unreacted peptide linkers using centrifugal filters (10 kDa MWCO). The number of pSer residues conjugated to eOD was quantified using a Malachite Green Phosphoprotein Phosphate Estimation Assay Kit (Thermo Scientific) against a standard curve of maleimide‐pSer linker. Signal from pSer‐eOD was compared to the background from an unconjugated eOD antigen control. Labeled proteins were prepared using NHS‐Alexa Fluor dyes (AF568, AF647 and AF750; Thermo Fisher) by reaction of 6 eq. fluorophore with either eOD (1 mg mL^−1^) or IL‐21 fusion protein (1 mg mL^−1^) in phosphate buffered saline for 20 min at RT and purified using centrifugal filtration (10 kDa MWCO).

### Cloning, Protein Purification, and Phosphorylation Analysis

The sequences for IL‐21‐Alb‐fusion protein were derived from the amino acid sequence of recombinant murine IL‐21 (catalog 210‐21; Peprotech) and fused to mouse serum Alb as previously described.^[^ ^23^, ^31^ ^]^ ABP was cloned into the IL‐21‐Alb‐fusion protein construct at the C‐terminus by In‐Fusion (Takara Bio Inc.) followed by poly‐Histidine (His) tags for purification.^[^ ^23^ ^]^ Human cDNA from Fam20C (Horizon, previously DharmaCon) was also cloned into gWiz with a terminal KDEL tag (without a His tag). All plasmids were transformed into Stellar Competent Cells (Takara Bio Inc.) and purified using the NucleoBond Xtra Maxi EF endotoxin‐free maxi prep kit (Takara Bio Inc.).

For protein production, plasmids were transiently transfected into HEK293‐F cells (1 mg total DNA/L cell culture) via polyethylenimine (2 mg L^−1^ cell culture) using the Freestyle 293 Expression system (Gibco). For all cotransections, cytokine plasmid:Fam20C plasmid transfection mass ratios were set at 9:1. His‐tagged proteins from cell culture supernatants were then purified using HisPur Ni‐NTA metal affinity resin (Thermo Fisher Scientific). Monomeric phosphorylated proteins were further purified using a custom anion exchange chromatography salt gradient (0/25/30/40/100% incremental gradient increase as depicted in Figure S1c, Supporting Information) on HiTrap Q HP columns attached to an AKTA FPLC system (Cytiva Life Sciences, formerly GE Healthcare). Proteins were buffer exchanged into Tris‐Buffered Saline (1×, Sigma‐Aldrich) using Amicon Ultra Centrifugal spin filters (10 kDa MWCO; Millipore Sigma). Purified proteins were confirmed to have low endotoxin levels (<0.1 EU per dose) by the Endosafe Nexgen‐PTS system (Charles River) and validated for size by SDS‐PAGE; phosphorylation was assessed by using the Malachite Green Phosphoprotein Phosphate Estimation Assay Kit (Thermo Scientific).

### Antigen:Cytokine Alum Binding and Release

Alum binding experiments were performed with fluorescently labeled proteins. A mass ratio of 10:1 alum:protein was used for all binding experiments and immunizations. For alum binding experiments conducted with cytokine, a mass ratio of 10:6:1 alum:cytokine:protein was used. For binding assays, the labeled antigen was first incubated with Alhydrogel for 30 min in PBS pH 7.4 at 25 °C to allow binding, followed by the addition of the labeled cytokine for 30 min in PBS, then mouse serum was added for a final concentration of 10% v/v. Protein, alum, and serum mixtures were incubated for 24 h at 37 °C on a tube rotator, and solutions were centrifuged at 10 000 × *g* for 10 min to pellet alum. The concentration of unbound protein in the supernatant was then measured by fluorescence. Fluorescence measurements were performed using a Tecan Infinite M200 Pro absorbance/fluorescence plate reader. The fluorescence intensity was normalized to the total fluorescence of a sample that underwent the same processing but lacked alum.

### Whole Mouse Imaging of Vaccine Drainage

Mice were immunized s.c. at the tail base with fluorescently labeled antigen (eOD; labeled with AF750) and immunostimulatory cytokine (IL‐21; labeled with AF647); the signals were measured using an in vivo imaging system (IVIS) fluorescence imaging system over time at the injection site. Total radiance was normalized to the maximal IVIS signal for the antigen and cytokine (day 7 and day 5, respectively), due to fluorophore quenching effects at earlier time‐points. For these experiments, alum (50 µg) was mixed with either 5 µg of Ser_4_‐eOD or pSer_4_‐eOD and 30 µg of either IL‐21‐Alb or IL‐21‐Alb‐ABP for 30 min at 25 °C, and then injected s.c. into groups of BALB/c mice. IVIS imaging was carried out on a PerkinElmer Xenogen Spectrum IVIS, and the signals were tracked and quantified using Living Image software.

### Immunohistochemistry of Lymph Nodes

Mice were immunized by subcutaneous injections with 5 µg AF568‐labeled antigen (either Ser_4_‐ or pSer_4_‐conjugated eOD), 30 µg of AF647‐labeled cytokine (either IL‐21‐Alb or IL‐21‐Alb‐ABP) and 50 µg alum. Inguinal LNs were isolated and fixed with 4% paraformaldehyde overnight, washed, and embedded in a 3 wt% low melting point agarose at 37 °C then allowed to cool and solidify on ice for 15 min. 100 µm sections were prepared using a Vibratome (Leica VT1000S) and suspended in ice cold PBS then transferred into a blocking solution containing 10% goat serum, 0.2% Triton‐X100, and 0.05% sodium azide overnight at 37 °C prior to immunostaining. For LN sections, the tissues were stained for CD35 (clone 8C12, BD) and Ki67 (clone SolA15, Thermo Fisher). Antibodies were used at 1:100 dilution in blocking buffer overnight at 37 °C, followed by washes with PBS 0.05% Tween and mounted on a glass slide with ProLong Diamond antifade mounting medium (Life Technologies) and stored at 4 °C until they were ready to be imaged.

### Confocal Microscopy, Image Processing, and Quantification

LN sections were imaged on a Leica SP8 laser scanning confocal microscope with a 25× water objective. All laser and channel settings were kept constant between the different samples to allow for direct comparison of fluorescent signals in the follicles and GCs. Images were then processed and quantified with Fiji software. To quantify the average size of GCs, 15 individual follicles were imaged and quantified, sampled from six dLNs (*n* = 3 mice per group). GCs were labeled with anti‐Ki67, which helped identify proliferating lymphocytes within the follicles that were restricted to the dark zone of the GC. Next, to quantify antigen and cytokine signals localized within the GC, Z‐stacks of the same 15 individual follicles were imaged and quantified from overlapping signals from the antigen and cytokine channels with the GC. The integrated intensity values were calculated for the given region of interest marked by the GC staining for each given follicle.

### Germinal Center and T Follicular Helper Responses

Mice were immunized subcutaneously at the tail base, and inguinal LNs were collected on day 14 post immunization. For GC analysis, cells were mechanically digested and strained through 70 µm filters prior to staining for GC B cell analysis, cells were stained for viability (Live/Dead Fixable Aqua, Thermo Fisher), CD3e (dye: BV711, clone 145‐2C11; BioLegend), B220 (dye: PE‐Cy7, clone RA3‐6B2; BioLegend), CD38 (dye: FITC, clone 90; BioLegend), and GL7 (dye: PerCP‐Cy5.5, clone GL7; BioLegend), and for antigen‐specific staining biotinylated eOD conjugated to streptavidin‐BV421 and streptavidin‐PE (BioLegend). For T_FH_ analysis, cells were stained for viability (Live/Dead Fixable Aqua, Thermo Fisher) and against B220 (dye: BV510, clone RA3‐6B2; BioLegend), CD4 (dye: FITC, clone GK1.5; BioLegend), CD44 (dye: PE‐Cy7, clone IM7; BioLegend), PD‐1 (dye: BV421, clone RMP1‐30; BD Biosciences), and CXCR5 (dye: PE, clone 2G8; BD Biosciences). Samples were analyzed by flow cytometry on a BD Celesta and analyzed on FlowJo.

### ELISAs Analysis of Antibody Titers

Serum was collected from mice retro‐orbitally using microhematocrit capillary tubes and stored at −20 °C until analysis. To measure serum IgG titers against eOD, Nunc MaxiSorp plates (Invitrogen) were directly coated with unmodified, monomeric eOD (1 µg mL^−1^) for 4 h at 25 °C in PBS and blocked with 2% BSA in PBS overnight at 4 °C. Plates were washed with 0.05% Tween‐20 containing PBS, followed by the addition of serial dilutions of sera to the blocked plates for 2 h at 25 °C. Plates were washed again with 0.05% Tween‐20 in PBS, and incubated with goat anti‐mouse IgG‐HRP at 1:5000 dilution (BioRad) for 1 h at 25 °C. ELISA plates were washed and developed with TMB substrate then stopped with 2 n sulfuric acid prior to reading the absorbance values at 450 nm on a plate reader. For antibody isotype ELISAs, the same protocol described above was followed but with the following secondary antibodies; goat anti‐mouse IgG1 HRP (Invitrogen, A10551), and goat anti‐mouse IgG2a HRP (Invitrogen, M32207) or goat anti‐mouse IgG2b HRP (Invitrogen, M32407) at 1:2000 dilution.

To calculate the area under the curve (AUC) values for the ELISA analysis, the serum dilution factor was converted into log_10_ values for each of the individual raw absorbance curve values, followed by the AUC analysis on Prism. Once the AUC values were calculated for each individual absorbance curve, those values were then plotted to run statistical analysis between experimental groups.

### ELISPOT Analysis

Bone marrow ELISPOTs were performed in mice 3 months after immunization according to the manufacturer protocol (MabTech, catalog no. 3825‐2A). Briefly, 96‐well PVDF ELISPOT plates (MSIPS4510; Millipore Sigma) were coated with anti‐mouse IgG at 15 µg mL^−1^ in sterile PBS overnight at 4 °C. Cells were isolated from the femur and tibia of mice, ACK lysed, and 70 µm filtered in complete media (RPMI 1640 containing 10% FBS, 100 U mL^−1^ penicillin‐streptomycin, and 1 mm sodium pyruvate). The next day, plates were blocked with complete media for at least 30 min prior to adding cells with technical duplicates per mouse. For total IgG and antigen‐specific IgG, 50 000 and 250 000 cells were added per well, respectively, and incubated at 37 °C with 5% CO_2_ for 16 h. Plates were then washed with PBS. Antigen‐specific responses were determined by adding 1 µg mL^−1^ biotinylated eOD in PBS with 0.5% BSA to each well for 2 h at 25 °C. Total IgG responses were determined by adding 1 µg mL^−1^ anti‐mouse IgG‐biotin detection antibody in PBS with 0.5% BSA to each well for 2 h at 25 °C. Plates were washed again in PBS and incubated with 1:1000 streptavidin‐alkaline phosphatase in PBS with 0.5% BSA for 1 h at 25 °C. After washing, plates were developed with BCIP/NBT substrate (MabTech, catalog no. 3650‐10) and developed for 20 min, quenched with H_2_O, and dried prior to quantification on an ImmunoSpot CTL Analyzer.

### Physicochemical Characterization

Transmission electron microscopy and dynamic light scattering were used to analyze physicochemical properties of particles. 10 µL of premixed alum/IL21 or alum alone was dropped on 200 mesh copper grids coated with a continuous carbon film and dried at room temperature. Grids were then mounted on a single tilt holder equipped in the FEI Tecnai Spirit microscope. The microscope was operated at 120 kV and with a magnification in the range of ≈6000–40 000 for assessing particle size and distribution. The hydrodynamic size and zeta potential were assessed using a Malvern Zetasizer.

### Statistical Analysis

Statistics were analyzed using GraphPad Prism software. All graphs represented mean and standard deviations. Comparisons of more than two groups were performed using a one‐way ANOVA with a Tukey's post‐test to determine statistical significance. For comparisons of experiments with only two groups, two‐tailed Mann–Whitney *U*‐test was used. *p* values <0.05 were determined to reach statistical significance.

## Conflict of Interest

D.J.I., Y.A., and T.J.M. are inventors on patent applications related to the technology described in the manuscript.

## Supporting information



Supporting Information

## Data Availability

The data that support the findings of this study are available from the corresponding author upon reasonable request.
