# Supplementary material for: Co‐Anchoring of Engineered Immunogen and Immunostimulatory Cytokines to Alum Promotes Enhanced‐Humoral Immunity
Source: Adv Ther (Weinh). 2022 Apr 7;5(7):2100235. doi: 10.1002/adtp.202100235 (PMC9595138; doi:10.1002/adtp.202100235)
Supplement: Supplementary file 1 — Supporting Information [file ADTP-5-2100235-s001.pdf]

ADVANCED THERAPEUTICS

Supporting Information

for *Adv. Therap.*, DOI 10.1002/adtp.202100235

Co-Anchoring of Engineered Immunogen and Immunostimulatory Cytokines to Alum Promotes Enhanced-Humoral Immunity

*Jason Y. H. Chang, Yash Agarwal, Kristen A. Rodrigues, Noor Momin, Kaiyuan Ni, Benjamin J. Read, Tyson J. Moyer, Naveen K. Mehta, Murillo Silva, Heikyung Suh, Mariane B. Melo, K. Dane Wittrup and Darrell J. Irvine**

Supporting Information

Co-delivery of engineered immunostimulatory cytokine and immunogen to alum promotes enhanced humoral immunity

*Jason Y.H. Chang, Yash Agarwal, Kristen A. Rodrigues, Noor Momin, Kaiyuan Ni, Benjamin J. Read, Tyson J. Moyer, Naveen Mehta, Murillo Silva, Heikyung Suh, Mariane B. Melo and Darrell J. Irvine**

Dr. J. Y. H. Chang, Y. Agarwal, K. A. Rodrigues, K. Ni, N. Momin, Dr. B. J. Read, Dr. T. J. Moyer, Dr. N. Mehta, Dr. M. Silva, H. Suh, Dr. M. B. Melo, Prof. K. Dane Wittrup, Prof. D. J. Irvine
Koch Institute for Integrative Cancer Research, Massachusetts Institute of Technology,
500 Main Street, Cambridge, MA 02142, UA
Email: djirvine@mit.edu

Dr. J. Y. H. Chang, K. A. Rodrigues, Dr. B. J. Read, Dr. T. J. Moyer, Dr. M. Silva, Dr. M. B. Melo, Prof. D. J. Irvine
Ragon Institute of Massachusetts General Hospital, Massachusetts Institute of Technology and Harvard University, Cambridge, MA 02139, USA

K. A. Rodrigues, Dr. B. J. Read
Harvard-MIT Health Sciences and Technology Program, Institute for Medical Engineering and Science, Massachusetts Institute of Technology, Cambridge, MA 02139, USA.

Y. Agarwal, K. A. Rodrigues, N. Momin, Dr. B. J. Read, Dr. N. Mehta, Prof. K. Dane Wittrup, Prof. D. J. Irvine
Department of Biological Engineering, Massachusetts Institute of Technology, Cambridge, MA 02139, UA

Prof. K. Dane Wittrup
Department of Chemical Engineering, Massachusetts Institute of Technology Cambridge, MA 02139, USA

Prof. D. J. Irvine
Department of Materials Science and Engineering, Massachusetts Institute of Technology Cambridge, MA 02139, USA

K. A. Rodrigues, Prof. D. J. Irvine
Consortium for HIV/AIDS Vaccine Development, The Scripps Research Institute, La Jolla, CA 92037, USA.

Prof. D. J. Irvine
Howard Hughes Medical Institute, Chevy Chase, MD 20815, USA

[+] Y. Agarwal, K. A. Rodrigues and N. Momin contributed equally to this work

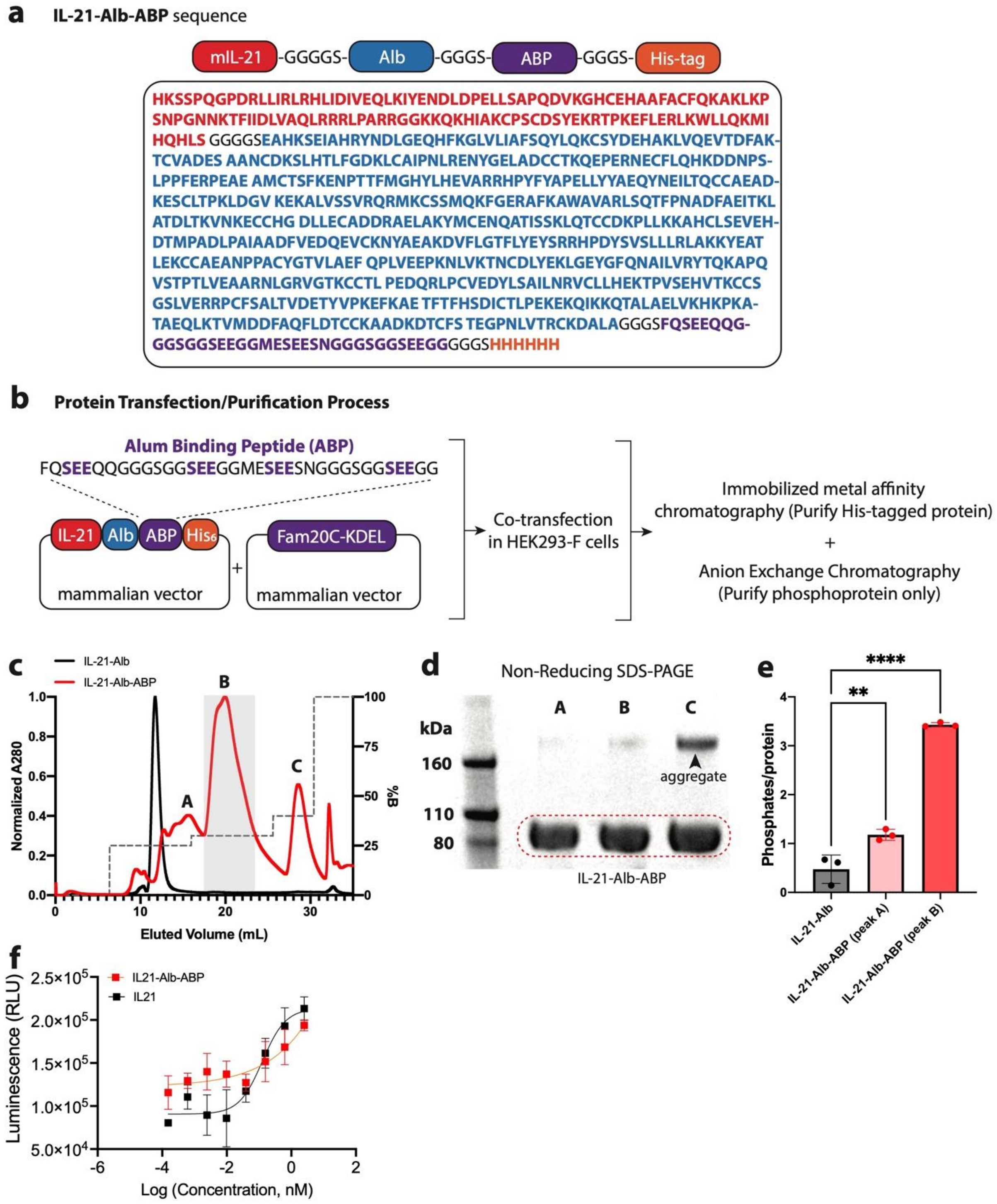

Figure S1. **Characterization of IL-21-Alb-ABP and pSer-modification of eOD antigen to facilitate anchoring to alum**. a) Co-expression of IL-21-Alb-ABP and Fam20C-KDEL enables in-cell site-specific serine phosphorylation of the cytokine. b) Representative trace of anion exchange chromatography of IL-21-Alb-ABP (blue trace) to separate phosphorylated cytokine compared to IL-21-Alb (non-phosphorylated cytokine; red trace). c) Collected protein peaks were run on SDS-PAGE gel stained with Coomassie Blue to identify both the correct molecular weight (~85.8 kDa) and monomeric protein (peaks A and B). d) Phosphorylation of IL-21-Alb-ABP were determined with a malachite green assay to estimate the number of phosphoserine residues per protein. e) Chemical structure of pSer peptide-linker for site-specific antigen modification. pSer-conjugated eOD and IL-21-Alb-ABP are

anchored to alum via ligand exchange between the phosphates in the phosphoserine residues and hydroxyls on the surface of alum. f) IL-21 protein potency evaluated by incubation of ANBL-6 cells with indicated concentrations of IL-21 or IL-21-Alb-ABP for 3 days, followed by assessment of total cell proliferation by Cell titer Glo luminescence. Statistical significance was determined by one-way ANOVA followed by Tukey's post hoc test. ns, not significant; $^{**}p<0.01$; $^{****}p<0.0001$. Values plotted are means ± standard deviation.

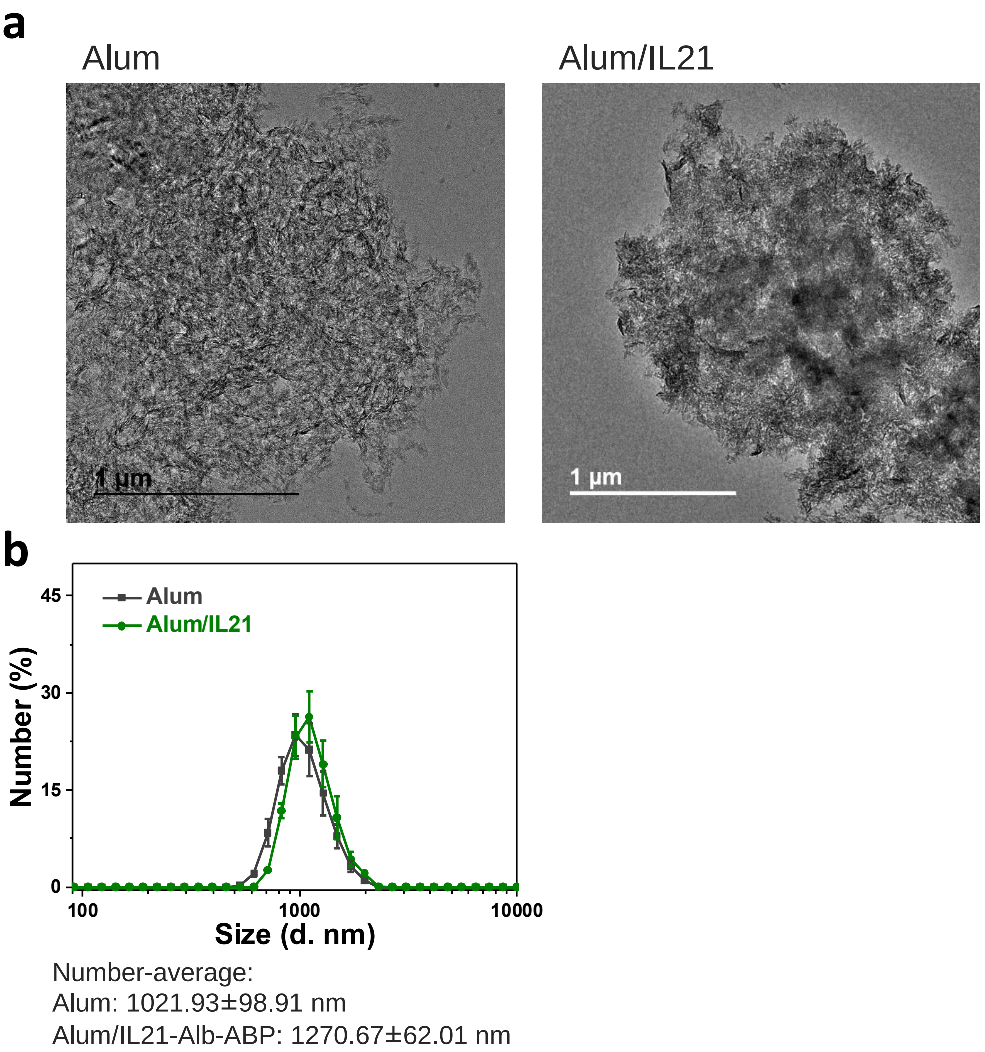

Figure S2. Physical characterization of alum/IL-21-Alb-ABP complexes. a) TEM imaging of neat Alhydrogel (left, denoted as Alum) or alhydrogel loaded with IL-21-Alb-ABP (right, denoted as Alum/IL21). b) Hydrodynamic size analysis of the particle size distribution of neat alum, or alum loaded with IL-21-Alb-ABP.

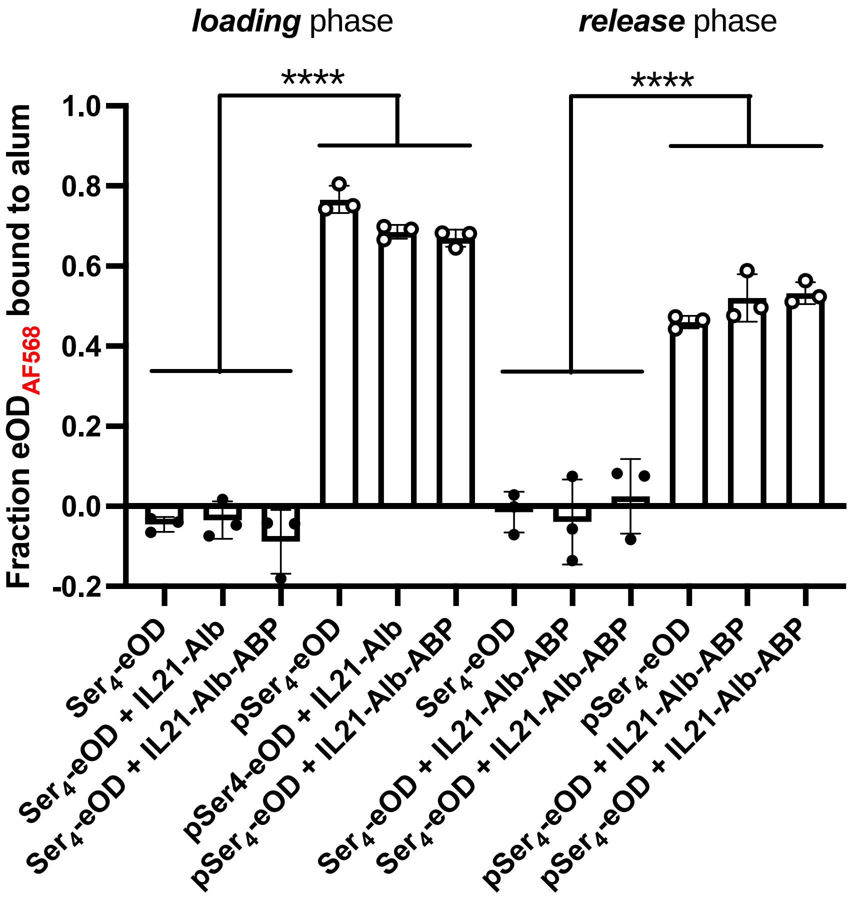

Figure S3. IL-21-Alb-ABP and eOD co-adsorption to alum. Fluorescently labeled eOD proteins with Alexa Fluor 568 (either Ser_4- or $pSer_4$-conjugated; at 10µg mL^{-1}) were mixed with Alhydrogel (100µg mL^{-1}) for 30 mins, followed by sequential loading of unlabeled IL-21 fusion proteins (either IL-21-Alb or IL-21-Alb-ABP; at 10µg mL^{-1}) for 30 mins, then incubated in PBS containing 10% mouse serum for 1h, followed by fluorescence spectroscopy to measure eOD protein remaining bound to alum with and without the presence of IL-21 fusion proteins.

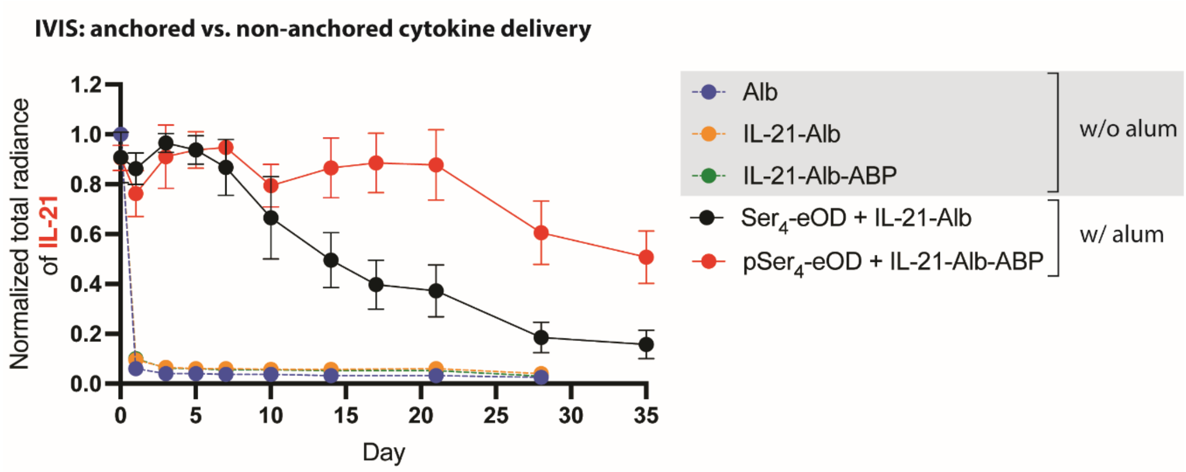

Figure S4. **IVIS imaging of alum-anchored cytokines vs. non-anchored cytokines**. a) Non-anchored fluorophore-labeled albumin protein (Alb), IL-21-albumin fusion (IL-21-Alb), IL-21-Alb-ABP were injected without alum present s.c. in BALB/c mice (n=4 animals per group). This was compared to anchored proteins, were each protein were fluorescently labeled (IL-21-Alb or IL-21-Alb-ABP; 30µg of protein) and mixed with Alhydrogel (50µg of alum), and injected s.c. in BALB/c mice (n=4 animals per group) followed by longitudinal whole animal in vivo imaging system (IVIS) imaging of fluorescence at the injection sites.

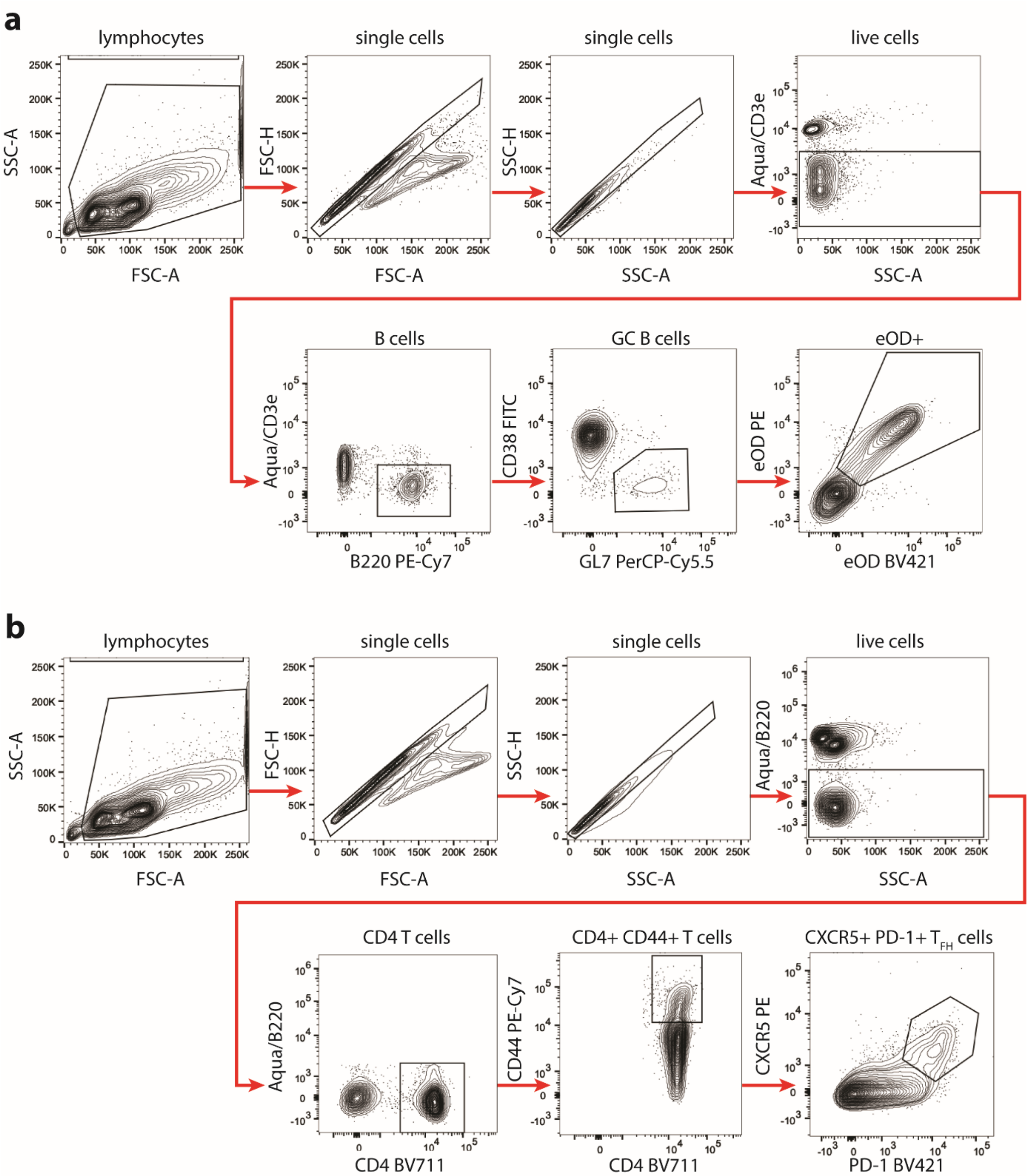

Figure S5. Flow cytometry gating. a) Representative flow plots of eOD-specific germinal center (GC) B cells, and b) T follicular helper (T_{FH}) cells.

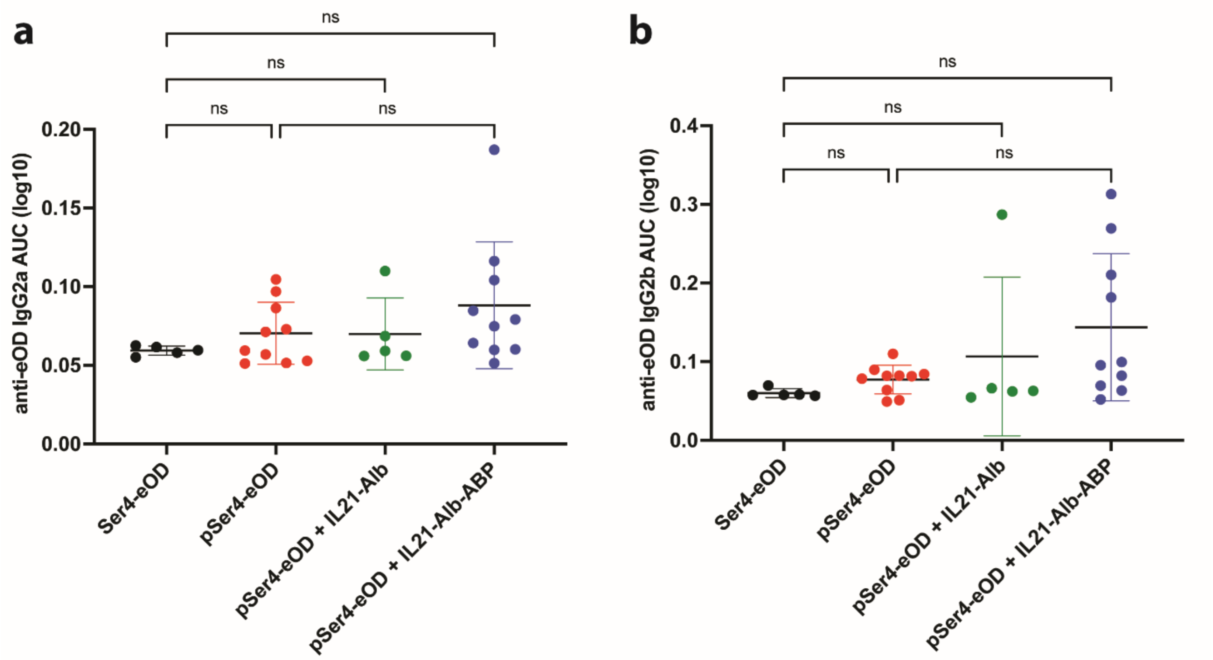

Figure S6. Antibody isotypes were assessed at week 6 for IgG2a (a) and IgG2a (b).
